# Massive Transfusion Increases Serum Magnesium Concentration

**DOI:** 10.3390/jcm12155157

**Published:** 2023-08-07

**Authors:** Justyna Malinowska, Milena Małecka-Giełdowska, Katarzyna Pietrucha, Gabriela Górska, Dagmara Kogut, Olga Ciepiela

**Affiliations:** 1 Department of Laboratory Medicine, Medical University of Warsaw, 02-097 Warsaw, Poland; milena.malecka@wum.edu.pl; 2 Doctoral School, Medical University of Warsaw, 02-091 Warsaw, Poland; 3 Central Laboratory, Central Teaching Hospital of University Clinical Center, Medical University of Warsaw, 02-097 Warsaw, Poland; 4 Students Scientific Group of Laboratory Medicine, Medical University of Warsaw, 02-097 Warsaw, Poland

**Keywords:** magnesium, hypomagnesemia, transfusion, RBC

## Abstract

(1) Background: The massive transfusion of packed red blood cells (RBCs) is a lifesaving procedure, but it is associated with complications, e.g., dysmagnesemia. Since magnesium is an intracellular ion, the transfused RBCs can significantly influence the magnesium concentration in the recipient’s blood. (2) Methods: A retrospective study was performed among 49 patients hospitalized in the Central Clinical Hospital of the Medical University of Warsaw who received a massive blood transfusion (≥4 units/h). Data on laboratory results and patient history were collected from the hospital database. The intracellular RBCs magnesium concentration was measured in 231 samples using the colorimetric method. (3) Results: There were statistically significant changes in the mean serum magnesium concentration preoperatively and 24 h postoperatively (0.87 ± 0.13 vs. 1.03 ± 0.14, *p* < 0.00001) and 48 h postoperatively (0.87 ± 0.13 vs. 1.06 ± 0.15, *p* < 0.00001). Patients who died had significantly higher serum magnesium concentrations (*p* < 0.05). The median intracellular magnesium concentration in RBCs was 0.91 (0.55–1.8) mmol/L, which is below the reference values of 1.65–2.65 mmol/L. (4) Conclusions: Transfused RBCs significantly increased the serum magnesium concentration 24 h and 48 h postoperatively. It could be a result of mild hemolysis, as the median intracellular magnesium concentration in RBCs was below the reference values.

## 1. Introduction

Massive transfusions of red blood cells (RBCs) are performed on patients in hemorrhagic shock and with other life-threatening conditions [1]. Although their use is vital for patients to survive, it is associated with numerous serious health problems such as hypothermia, coagulopathies, and metabolic and electrolyte disturbances [2,3,4]. The last may result from previously existing disorders in the patient or blood donor, changes in blood products resulting from their prolonged storage, or a loss of minerals related to the method of blood product preparation [5]. To avoid clotting, the blood is collected into bags containing the anticoagulant citrate phosphate dextrose (CPD) [6]. Citrate is a magnesium chelator that lowers the extracellular concentration of this mineral [7].

Electrolyte disorders are a common massive transfusion complication; however, it is not known how often they are caused by dysmagnesemia in blood products. For severely ill patients, magnesium balance disturbances are commonly diagnosed already on hospital admission, seemingly increasing the importance of the high quality of blood products [8]. One of the main reasons for magnesium imbalances is chronic kidney disease (CKD), as kidney function is vital for proper magnesium handling [9]. However, it is not until stages 4 and 5 of CKD that those abnormalities, mostly hypermagnesemia, are visible in laboratory test results because of the compensatory mechanisms [10]. Although hypermagnesemia is thought to be rare, it is an important in-hospital mortality risk factor [11,12]. Moreover, decreased serum magnesium levels, which occur in 9–15% of critically ill patients, are also associated with an increased risk of death [8,13]. Hypomagnesemia is a common electrolyte disturbance occurring after severe events, like cardiac arrests [14]. However, it also seems to be one of the disturbances following surgeries in general and cardiac surgery, after which magnesium levels fall within the first 24 h [15,16].

Magnesium homeostasis disorders in hospitalized patients may be a result of the drugs and procedures applied. Among the drugs most commonly used in severely ill patients, diuretics and antimicrobials have the biggest impact on magnesium metabolism, as they increase its renal excretion. Moreover, medications taken by patients for chronic illnesses, e.g., cisplatin, calcineurin inhibitors, cetuximab, panitumumab, matuzumab, proton-pump inhibitors, foscarnet, cardiac glycosides, and loop and thiazide diuretics, cause magnesium deficiency [8,17,18]. Continuous veno–venous hemodiafiltration (CVVHDF) also decreases the serum magnesium concentration because the citrate used in this procedure in order to prevent blood from clotting is also a magnesium chelator [19,20].

Magnesium disturbances also occur in healthy people, some of whom decide to donate blood. According to WHO, 75% of US citizens do not meet their dietary magnesium requirements [4]. Therefore, it is likely that magnesium deficiency is common in healthy subjects, although difficult to detect in laboratory tests, as only 0.3% of magnesium is in the extracellular space, e.g., serum [21,22]. Moreover, the intracellular magnesium concentration in packed RBCs seems to reflect the body’s magnesium status more precisely than the serum magnesium concentration [23]. Because of the fact that magnesium is an intracellular ion, the transfused red blood cells (RBCs) can significantly influence the magnesium concentration in the recipient’s blood. Furthermore, the volume of transfused blood products is correlated with the observed change in the serum magnesium concentration [24,25].

This study aims to assess how the massive transfusion of packed red blood cells (RBCs) influences the patients’ postoperative serum magnesium concentration and how often blood products have abnormal intracellular magnesium concentrations.

## 2. Materials and Methods

A retrospective study was performed among 49 patients hospitalized in the Central Clinical Hospital of the Medical University of Warsaw between November 2021 and March 2023 who received a massive blood transfusion. Massive transfusions were defined as transfusions with ≥four units during a single hour. The cut-off point is based on the observations of other studies and adopted due to the lack of a universally accepted massive transfusion definition [26,27]). The median volume of transfused packed RBCs was 2600 mL (2350; 2950). The size of the study group was determined based on the study of Chrun et al. (power (1-β err prob) = 0.95) [24]. The inclusion criteria were the serum magnesium concentration measured preoperatively on admission and 24 h or 48 h postoperatively and the preserved samples of transfused red blood cells (RBCs). The exclusion criteria were data gaps and a history of prior blood transfusions. There were no acute posttransfusion reactions, neither immunological nor non-immunological, including hemolysis, in any of the patients included in the study.

The study was conducted in accordance with the rules of the Bioethical Committee of the Medical University of Warsaw, and the data were anonymized.

The data on the serum magnesium concentration preoperatively, 24 h postoperatively, or 48 h postoperatively, the potassium concentration 24 h postoperatively, the creatinine concentration, the eGFR, the reason for hospitalization, the drugs administered intravenously, chronic illnesses, and outcomes were collected from the hospital database.

Preparation of red blood cells: samples of packed RBCs for transfusion were centrifuged for 15 min at 1500× *g*. The preservative fluid was then separated, and a lysis buffer containing no Mg2+ ions with a pH of 9.4 was added to the remaining red blood cell concentrate in a 1:2 ratio. The samples were then homogenized and subjected to a magnesium concentration measurement. We also measured the total magnesium concentration in the separated preservative fluid.

The total magnesium in red blood cells as well as in serum and preservative fluid was measured using the colorimetric method. The reference values for the total serum magnesium for adults are in the range of 0.75–1.0 mmol/L. The reference values of the RBCs intracellular magnesium concentration are in the range of 1.65–2.65 mmol/L and were drawn from Costello et al.’s study [28]. The potassium concentration was measured with the indirect potentiometry method and adjusted mathematically to a standardized pH of 7.4. The reference values for potassium for adults are in the range of 3.5–5.1 mmol/L. The creatinine concentration was measured using the CRE2 assay in the biochemical Dimension system. The reference values for females are in the range of 0.48–0.93 mg/dL, and for males, they are in the range of 0.63–1.16 mg/dL [29]. The eGRF value was calculated from the CKD-EPI formula. The eGFR value was used to determine the stage of chronic kidney disease—respectively, stages 1–2, eGFR ≥ 60 mL/min/1.73 m^2^, stage 3, eGFR = 30–59 mL/min/1.73 m^2^, stage 4, eGFR = 15–29 mL/min/1.73 m^2^, and stage 5, eGFR < 15 mL/min/1.73 m^2^ [30].

The analyzers used to take measurements were the Cobas 702 (Hoffmann-La Roche AG, Basel, Switzerland), Dimension EXL (Siemens Healthineers, Erlangen, Germany), and ABL 875 FLEX analyzer by Radiometer (Copenhagen, Denmark). For the Dimension EXL analyzer, the measurement range was 0.0–20.0 mg/dL (0.0–8.22 mmol/L), and the precision was high (coefficient of variation (CV) 1.7–1.9%). For the Cobas 702 analyzer, the measurement range was 0.10–2.0 mmol/L (0.243–4.86 mg/dL), and the precision was also high (coefficient of variation (CV) 0.7–1.3%).

Statistical Analysis was performed using Microsoft Office Excel 2019 and Statsoft Statistica 13.3. The tests used were: a *t*-Student test, Mann–Whitney test, ANOVA test, Chi-squared test, and Spearman’s correlation coefficient. The Shapiro–Wilk test was used to assess the normality of the result distribution. A *p*-value < 0.05 was considered as statistically significant.

## 3. Results

The mean age of the patients included in the study was 55.7 ± 16.2 years, and 20 of them (41%) were female. The majority of the study group (80%, n = 39) consisted of patients operated on for various reasons (heart or aortic defect, cancer, organ transplant, multi-site trauma). The rest (20%, n = 10) consisted of patients that needed packed RBCs transfusion due to severe anemia or bleeding. Only 10% (n = 5) of the study group did not have chronic illnesses, and 27% (n = 13) of the patients died. For basic information on the patients, see Table 1.

Preoperative magnesium concentration abnormalities, although present in 15 patients (31%), were not severe. Patients with hypomagnesemia (n = 7, 14%) had a mean serum magnesium concentration of 0.67 ± 0.03 mmol/L, while patients with hypermagnesemia (n = 8, 16%) had one of 1.06 ± 0.05 mmol/L.

The influence of factors that could potentially affect magnesium homeostasis such as undergoing a surgical procedure, comorbidities, drugs administered intravenously, and dialysis on the serum magnesium concentration was analyzed. Statistically significant associations were found only for sedatives and antidiuretics administration. The mean serum magnesium concentration was higher in patients who received sedatives (respectively, 1.07 vs. 0.98, *p* < 0.05, and 1.11 vs. 1.02, *p* < 0.05) as well as in patients who received diuretics (1.105 vs. 1.00, *p* < 0.05, 1.15 vs. 1.04, *p* < 0.05). The results are summarized in Table 2.

**Figure 1:**
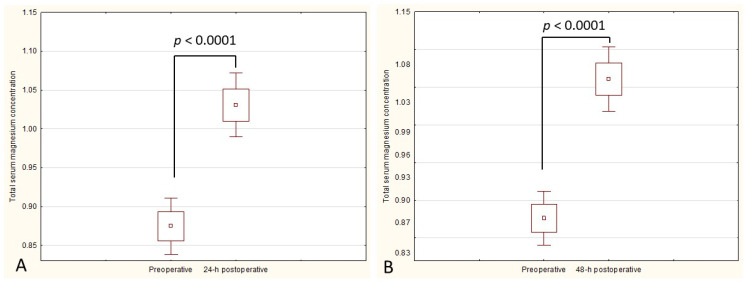
(**A**) The difference in the total serum magnesium concentration between preoperative and 24 h postoperative values (*p* < 0.00001). (**B**) The difference in the total serum magnesium concentration between preoperative and 48 h postoperative values (*p* < 0.00001).

The mean preoperative serum magnesium concentration was significantly lower than 24 h postoperative (0.87 ± 0.13 vs. 1.02 ± 0.14, *p* < 0.00001) and 48 h postoperative (0.87 ± 0.13 vs. 1.06 ± 0.15, *p* < 0.00001) values (Figure 1A,B). There was a strong correlation between the postoperative serum magnesium concentration and the volume of transfused packed RBCs (R = 0.66, *p* < 0.05).

**Figure 2:**
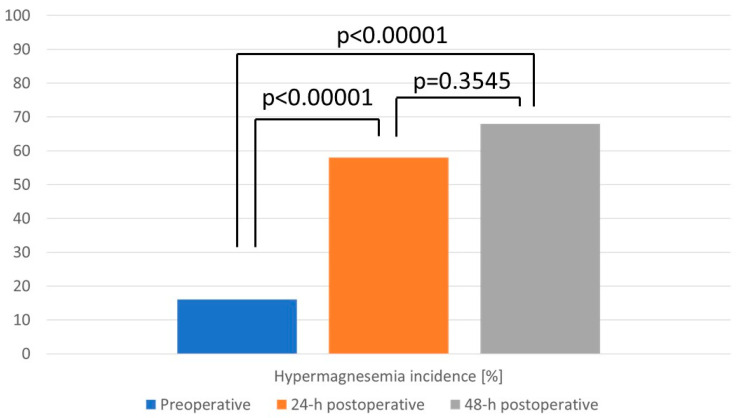
Differences in the hypermagnesemia incidence before the massive transfusion and 24 h and 48 h postoperatively.

Moreover, the incidence of hypermagnesemia increased significantly after a blood transfusion from 16% (n = 8) of the study group up to 57% (n = 28) 24 h postoperatively and 67% (n = 33) 48 h postoperatively (Figure 2).

**Figure 3:**
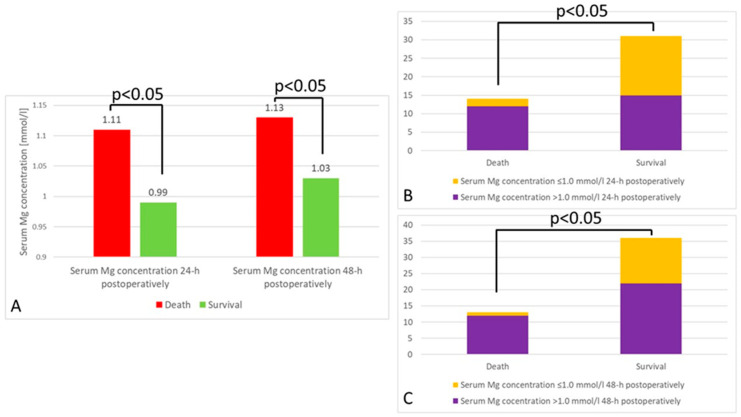
(**A**) Mean serum magnesium concentration depending on the outcome 24 h and 48 h postoperatively (*p* < 0.05). (**B**) The difference in hypermagnesemia prevalence between patients who died and survived 24 h postoperatively (*p* < 0.05). (**C**) The difference in hypermagnesemia prevalence between patients who died and survived 48 h postoperatively (*p* < 0.05).

There was no association between the preoperative serum magnesium concentration and the outcome (*p* = 0.13). However, there were statistically significant associations between the 24 h and 48 h postoperative serum magnesium concentrations and the outcome. In both cases, patients who died had higher serum magnesium concentrations (Figure 3A). Moreover, hypermagnesemia 24 h and 48 h postoperatively was found significantly more often in patients who died (Figure 3B,C).

As kidney function influences the serum magnesium concentration, data on the creatinine and eGFR of patients included in the study were collected. Increased creatinine levels were common, occurring in 60% of the female patients (n = 12), with a median of 1.40 (0.94, 4.49) mg/dL, and in 59% of male patients (n = 17), with a median of 1.86 (1.17, 4.02) mg/dL. However, there was no statistically significant association with sex (*p* = 0.24). Since only a few patients bleeding at admission were reported, an increased creatinine concentration in the majority of patients was not associated with hypovolemia.

Despite the increased creatinine levels among the patients included in the study, the majority of our study group (84%, n = 41) had an eGFR in a range of one to three stages of CKD, and only one-fifth (n = 8) had advanced CKD (stages 4–5).

However, there was a statistically significant average negative correlation between the preoperative magnesium concentration and eGFR R = −0.34 (*p* < 0.05). On the other hand, there was no statistically significant correlation between the preoperative magnesium and creatinine concentrations (R = 0.26, *p* = 0.07). Furthermore, no statistically significant correlation was found between either the total serum magnesium concentration 24 h postoperatively and creatinine (R = 0.24, *p* = 0.1077) or eGFR (R = −0.24, *p* = 0.1), nor was there one between the serum magnesium concentration 48 h postoperatively and creatinine (R = 0.26, *p* = 0.79) or eGRF (R = −0.28, *p* = 0.55).

There was also no statistically significant difference in the mean preoperative, 24 h, and 48 h postoperative serum magnesium concentrations between the CKD stages, respectively (*p* = 0.68, *p* = 0.0675, *p* = 0.0538). Among patients that had an abnormal preoperative serum magnesium concentration and advanced CKD with an eGFR lower than 29 (stages four to five), only one had hypermagnesemia.

The intracellular magnesium concentration was measured in 231 packed RBC samples. The median intracellular magnesium concentration in packed RBCs was 0.91 (0.55–1.8) mmol/L, which is below the reference values of 1.65–2.65 mmol/L (18), whereas the mean magnesium concentration in the preservative fluid was 0.214 ± 0.036 mmol/L. Magnesium levels below the reference values range were found in 198 (86%) samples, with a median concentration of 0.9 (0.24–1.62) mmol/L; normomagnesemia was found in 32 (14%) samples, with a median concentration of 1.95 (1.66–2.4) mmol/L. There were no samples with hypermagnesemia.

No correlation between the mean intracellular magnesium concentration in transfused RBCs and the change in the serum magnesium concentration 24 h (R = 0.24, *p* = 0.1) and 48 h (R = 0.008, *p* = 0.95) (Spearman’s correlation coefficient) postoperatively was found.

To confirm that the observed changes in the serum magnesium concentration were not due to noninfectious transfusion-associated adverse events, potassium levels preoperatively and 24 h postoperatively were also analyzed. The mean potassium concentration in our study group was 3.98 ± 0.6 mmol/L preoperatively and 4.47 ± 0.67 mmol/L 24 h postoperatively. The hypokalemia prevalence decreased postoperatively from 22% (n = 11) to 12% (n = 6) of the study group, whereas the hyperkalemia prevalence increased from 2% (n = 1) to 20% (n = 10) of the enrolled patients. Out of those patients, 10 had hypermagnesemia. There was no association between hypermagnesemia occurrence and serum potassium concentration either 24 h postoperatively (*p* = 0.18) or 48 h postoperatively (*p* = 0.36).

## 4. Discussion

In our study, preoperative serum magnesium concentration disturbances were found in 31% of patients, with hypermagnesemia being more common than hypomagnesemia—16% and 14% of patients, respectively. Hypermagnesemia, although thought to be rare, is a condition with an incidence of 1.78–10% in the hospital population [31,32]. However, it seems to be substantially more frequent in the ICU department, with the incidence reaching 39.8% [33]. On the other hand, hypomagnesemia is usually found in a larger proportion of hospitalized patients, 8.43–15%, which is in line with the incidence observed in our study group [31,32].

Maintaining magnesium homeostasis requires shifts of magnesium ions between the intracellular and extracellular spaces [34]. Serum magnesium levels are also tightly regulated by kidneys, intestines, and bones [33]. However, in critically ill patients, neither regulatory mechanisms nor insulin supply function properly. Additionally, organ dysfunctions are frequent [34]. Improper kidney function is one of the main causes of dysmagnesemia. Renal wasting results in hypomagnesemia, and decreased glomerular filtration causes hypermagnesemia. The latter can also be a result of cell and tissue damage, sepsis, hypothyroidism, Addison’s disease, or lithium treatment [33]. The former can be caused by gastrointestinal abnormalities or medicines, such as loop diuretics, thiazides, cisplatin, or proton pump inhibitors [8].

Although 90% of our study group was treated for chronic illnesses before hospitalization (the aforementioned diseases associated with a higher incidence of magnesium homeostasis disorders, among others), there was no association between the presence of comorbidities and the mean serum magnesium concentration at any time of the hospitalization. However, the serum magnesium concentration seems to be a better tool for observing sudden changes in the body than for the assessment of its magnesium status [12]. However, there were associations between the 24 h and 48 h postoperative serum magnesium concentrations and sedatives and diuretics intravenous administration. In both cases, patients who received medications had higher serum magnesium concentrations. Based on the literature data, sedative drugs do not affect magnesium homeostasis; however, not every patient in our study group received a sedative drug. Therefore, the study group may be too small to draw valid conclusions from this observation [35,36,37]. As for loop diuretics, e.g., furosemide, which was most commonly administered to patients in our study group, their use was also associated with an increased serum magnesium concentration in a study by Kieboom et al. [38].

There was no significant difference in the serum magnesium concentration between patients that were operated on and those that had massive RBCs transfusion administered because of other reasons. This is contrary to the observations of other researchers, who reported a decrease in serum magnesium concentrations after surgeries [8,15].

In our study group, 46% of patients had an eGFR of 60 or more, which can already mean CKD stage 1 (90–60 mL/min/1.73 m^2^) but is still associated with proper kidney function [39]. Furthermore, another 36% had CKD stage 3, and at this stage, compensatory mechanisms should maintain a normal serum magnesium concentration [10,40]. Among patients with advanced CKD (eGFR < 30), only one had hypermagnesemia, and no one had hypomagnesemia. Additionally, we did not find statistically significant differences in the serum magnesium concentration between patients at different stages of CKD, which is contrary to the results of Coburn et al. [41]. However, advanced CKD was not common in our study group, so we can only state that decreased glomerular filtration was not associated with an increased pre- or postoperative serum magnesium concentration in our study. Although around 60% of enrolled patients had increased creatinine levels, it is not the best indicator of kidney function, as an elevated creatinine concentration can be caused by a diet rich in animal protein, large muscle mass, and some medicaments [39]. Additionally, in most patients from our study group, the abnormal serum magnesium levels did not develop until post-transfusion.

Massive blood transfusion resulted in a significantly elevated incidence of hypermagnesemia both 24 h and 48 h postoperatively (*p* < 0.00001). Furthermore, there was a strong correlation between the volume of transfused packed RBCs and the serum magnesium concentration (R = 0.66, *p* < 0.05). This result is in stark contrast to those obtained by other researchers, who found a significant reduction in the concentration of magnesium in the serum of patients after blood transfusion [24,25]. Moreover, elevated magnesium levels were associated with the outcome. In our study, patients who died had significantly higher serum concentrations of magnesium both 24 h and 48 h postoperatively. Moreover, hypermagnesemia was more common in patients who died. This is in line with our earlier studies’ results but also with other researchers’ observations [11,33,42,43,44].

One plausible explanation is that the increased postoperative serum magnesium concentration was a result of hemolysis, as it would release magnesium ions. Hemolytic transfusion reactions are listed among noninfectious transfusion complications [45]. Acute hemolysis is always caused by an immune reaction and happens within 24 h of transfusion. However, it is a rather rare complication with an incidence of 1 to 5 per 50,000 transfusions. In our study group, hypermagnesemia affected 57% of patients 24 h postoperatively and 67% of patients 48 h postoperatively [46]. Acute complications associated with massive blood transfusion also include hypo- and hyperkalemia [2]. However, those disturbances were not common, as hypokalemia was found in 12% and hyperkalemia was found in 20% of the patients. Notwithstanding, hemolysis may be caused by other factors, such as fluid warmers [47]. The warming of fluids during transfusion is a procedure performed in order to avoid hypothermia, another blood transfusion complication. Blood warming is generally safe and causes only mild hemolysis, with no clinical impact on the patients [47]. Moreover, it would explain why the serum magnesium concentration increased postoperatively, even though hypomagnesemia was found in 86% of packed red cells.

An intracellular magnesium concentration below the reference values range is an indicator of a low body magnesium status [14]. Although it was believed for years that the intracellular magnesium concentration is stable, it is now known that the concentration of magnesium inside the cells can be influenced by hormones and other factors and even due to a decreased concentration in the blood serum, which causes the displacement of ions from the intracellular to the extracellular space [23,48]. While hypomagnesemia in RBCs can be a result of the low body magnesium status of a donor, it can also be caused by ex vivo changes due to the production process and storage [49]—for example, the usage of anticoagulants and additional solutions that extend the durability of the preparation [5]. All of those currently in use have an acidic pH (∼5.6–5.8), whereas the physiological pH of blood is 7.3. During the first days of storage, the buffering capacity of the RBC allows them to adjust the pH, but it does not last for long. The pH of RBC increases gradually to approximately 6.5 after 6 weeks of storage [50]. This lower pH value alters the generation of adenosine 5′-triphosphate (ATP), vital for RBC’s survival, and 2,3-diphosphoglycerate (2,3-DPG), needed for oxygen transportation. Furthermore, RBCs’ biochemistry changes in this altered environment, including mechanisms of ion and osmotic channels [51]. Another issue is that RBCs’ susceptibility to storage-related damage may be linked to a donor. Lifestyle components, like physical activity, diet, alcohol consumption, and smoking, but also age and sex, influence the RBCs quality [52,53,54,55].

Among the measures used to assess the RBCs quality, the main focus is on the refrigerator temperature, residual leucocyte counts, and visible hemolysis. Biochemical ones, like ATP, 2,3-DGP, potassium, and calcium levels, also have such applications but are not so widely used, as they require more specialized methods of measurement and do not reflect all storage-related changes [56]. On the other hand, the magnesium concentration in blood products is not measured at all. For that reason, the comparison of the obtained results with those of other researchers is not possible. However, if the mean magnesium concentration in the preservative fluid was 0.214 ± 0.036 mmol/L, magnesium ions probably had not shifted from the intracellular to the extracellular space, and the reason for hypomagnesemia in transfused RBCs could be the hypomagnesemia of the donors.

Hypomagnesemia in healthy people, as blood donors must be, is usually a result of an inadequate magnesium intake [57]. This can be partly explained by the fact that soils are less rich in this mineral, and therefore, the magnesium content of agricultural produce is lower than it used to be. In addition, some magnesium is lost through food processing, which makes it difficult to meet the demand for this mineral [58]. Another factor that could be the cause of common hypomagnesemia in healthy people who decided to donate blood is stress [59]. There is a concept of a magnesium and stress vicious circle, in which stress causes increased magnesium loss and, as a result, its deficiency, which in turn negatively impacts the body’s response to stressors [60]. This theory fits the fact that magnesium deficiency and stress affect an increasing part of developed societies, and the symptoms of both of these conditions are very similar, including fatigue, irritability, gastrointestinal disorders, and headaches [59]. However, the diagnosis of this disorder may be a matter of the cut-off points used, as there is still some uncertainty about the values that should be adopted when trying to assess the magnesium concentration in RBCs [23].

The lack of a correlation between the mean magnesium concentration in transfused RBCs and the change in the serum magnesium concentration 24 h and 48 h postoperatively could be a result of mild hemolysis, which is suspected to be the reason for the postoperatively increased serum magnesium concentration. The release of intracellular magnesium cations could temporarily increase the concentration of this mineral in the serum and mask the effect of mostly hypomagnesemic transfused red blood cells.

The study has certain strengths that are worth mentioning. First, to our knowledge, it is the first study to assess changes in the serum magnesium concentration after transfusion while assessing the magnesium concentration in packed RBCs that were used for transfusion at the same time. Second, there are limited data on abnormal intracellular concentrations of magnesium in healthy people, and our results might shed some light on this matter.

## 5. Conclusions

Massive blood transfusions significantly affect the serum magnesium concentration; however, it may rather be a result of mild hemolysis than an influence of the intracellular magnesium concentration in packed red cells. The postoperative serum magnesium concentration was higher, and hypermagnesemia was significantly more prevalent in patients who died. Therefore, it might be a parameter that should be monitored in patients after massive transfusions. Despite the common intracellular deficit of magnesium in packed red blood cells, its concentration does not influence magnesium levels in the extracellular fluid of transfused patients.

## Figures and Tables

**Table 1:** Basic information regarding patients, serum magnesium concentration, and transfused packed RBCs in the study group.

No.	Reason for Hospitalization	Drugs Administered Intravenously during Hospitalization	Dialysis	Chronic Diseases	Survival	Preoperative Mg Concentration	24 h Postoperative Mg Concentration	48 h Postoperative Mg Concentration	RBCs Approximate Volume [mL]	The Mean Intracellular Concentration in Transfused RBCs
1	Multi-site trauma	Propofol, sufentanil, tazocin	No	Alcohol dependence syndrome	Yes	0.83	1.07	1.04	2600	1.325
2	Tight aortic stenosis	Biotraxon, lenovor, amiodarone, furosemide	No	Hypertension	Yes	0.88	1.08	1.11	2350	0.85
3	Severe vitamin B_12_ deficiency anemia	-	Yes	p-ANCA systemic vasculitis, hypertension, chronic kidney disease	Yes	0.95	1.01	1.1	2950	1.424
4	Hemorrhage of the digestive tract	Midanium, norepinephrine, adrenalin	Yes	Alcohol dependence syndrome	No	0.96	1.07	1.09	2600	1.34
5	Laparotomy due to disseminated cancer	Levonor, morphine, furosemide, meronem	No	Nonalcoholic steatohepatitis, portal hypertension, hepatic encephalopathy, t2 diabetes mellitus, hypertension, obesity	Yes	0.78	0.83	0.94	2350	0.62
6	Liver transplant	No data	No data	Inflammatory bowel disease, hypothyroidism	Yes	0.81	0.99	0.82	2950	1.028
7	Liver retransplantation	Metronidazole	Yes	Renal failure, recurrent cholangitis	Yes	0.71	0.82	0.89	2600	0.55
8	Severe aortic stenosis	Furosemide, insulin, tramadol, propofol, sufentanil	No	No	Yes	0.77	1.08	1.14	2350	1.705
9	Ascending aortic aneurysm	Biofazolin, propofol, sulfentanyl, furosemide	No	Hypertension	Yes	1.15	1.42	1.4	2950	1.59333
10	Insufficiency of the main artery valve	Adrenaline, norepinephrine, dobutamine, levosimendan, propofol, midazolam, sufentanil, morphine, furosemide, heparin, coradrone, lignocaine, biotraxone, antithrombin III, PPI	No	Heart failure, ascending aortic aneurysm, permanent atrial fibrillation, hypertension	No	0.99	1.09	1.12	2350	1.525
11	Aortic valve disease and coronary artery disease	Propofol, sulfentanyl, furosemide, ebrantil	No	Hypertension, hyperlipidemia, t2 diabetes mellitus, COPD, heart failure	Yes	0.88	0.99	1.04	2350	1.16
12	Liver transplantation	Unfractionated heparin	No	No data	Yes	0.87	0.85	0.83	2600	1.74
13	Replacement of the aortic valve with the ascending aorta	Propofol, sulfentanyl, levonor, dobutamine	No	Aortic regurgitation, ischemic heart disease, heart failure, hypertension, thyroid nodular goiter	No	0.82	1.06	1.14	2350	0.86
14	Bleeding from esophageal varices	Terlipressin, PPI, K supplementation, insulin	No	Liver failure, pancreatic head tumor infiltrating the bile ducts	Yes	0.83	1.1	1.04	2350	1.475
15	Severe iron deficiency anemia	Clonazepam, furosemide	No	Severe microcytic anemia, hypertension, atherosclerosis	Yes	1.03	1.11	1.08	2400	1.58
16	Sideropenic anemia caused by recurrent bleeding from hemorrhoids	-	No	Hypothyroidism	Yes	0.9	1.02	0.98	2350	1.72
17	Alcoholic cirrhosis of the liver	Norepinephrine, anti-encephalopathic drugs, antibiotic, propofol, sufentanil	No	Alcohol dependence syndrome, esophageal varices	No	0.96	0.99	1.07	2350	1.38
18	Abdominal pain after subtotal gastrectomy for gastric adenoma	Propofol, sufentanil, antibiotic, furosemide, norepinephrine	No	Hypothyroidism, diverticular disease	No	0.76	1.31	1.2	2350	1.8
19	Dissecting aneurysm of the main artery	Propofol, morphine, noradrenaline, epinephrine, glypressin, polfilin, meronem, vancomycin	Yes	Loeys–Dietz syndrome, hypertension, asthma	No	0.81	1.04	1.1	2950	1.476
20	Pancreatic cancer disseminated	Hydrocortisone, clemastine	No	No	Yes	0.98	-	1.21	2350	0.585
21	Dissection of the ascending aorta	Tramadol, trifas, biofazolin	No	Hypertension, aortic regurgitation, hypothyroidism	Yes	1.04	1.18	1.32	2350	0.605
22	Iron deficiency anemia	Venofer	No	Hypertension	Yes	1.01	-	1.18	2400	1.04
23	Decompensated liver cirrhosis	-	No	Ovarian cancer, portal vein thrombosis, hemorrhoids, depression	Yes	0.68	0.88	0.72	2350	1.23
24	Severe nosebleed after the completion of chemotherapy for sarcoma of the hip	-	No	Hypothyroidism	Yes	0.98	1.03	0.88	2350	1.425
25	Mitral valve defect	Vancomycin, cordarone, concor cor amiodarone, trifas	No	Heart failure, ventricular arrhythmias, dyslipidemia,	Yes	0.93	0.95	1.0	2350	0.97
26	Cerebellar tumor	Tazocin, vancomycin, ceftriaxone, amikacin, analgesics	No	Hypertension, gastroesophageal reflux	No	1.04	1.08	-	2400	1.265
27	Alcoholic cirrhosis of the liver	Vancomycin, analgesics, anti-encephalopathic drugs, anti-coagulants	No	CKD	Yes	0.75	0.83	0.91	2350	0.985
28	Progressive liver damage	Propofol, norepinephrine, fluconazole	Yes	No	No	0.71	1.05	0.98	2950	1.46
29	Iron and vitamin B_12_ deficiency anemia	-	No	Hypertension, hyperlipidemia, impaired fasting glucose	Yes	0.85	1.01	0.92	2350	0.855
30	Hepatic failure in the course of PBC	Diuretics, norepinephrine	No	Pancreatic tail cyst, kidney cysts	Yes	0.87	1.21	1.21	2950	0.828
31	Liver transplant for secondary biliary cirrhosis	Diuretics, norepinephrine	No	No data	Yes	1.08	1.19	1.33	2950	0.872
32	Multiple myeloma, kappa light chain disease, ISS 3, D-S IIIB	-	Yes	Hypertension, CDK	Yes	0.88	1.04	0.87	2950	0.724
33	Inflammation of the bile ducts	Morphine, midanium, norepinephrine	No	Ulcerative colitis in remission	Yes	0.91	1.17	1.08	2950	0.888
34	Liver transplantation due to HCV infection	Sufentanil	No	Hypertension	Yes	0.85	0.98	1.07	2600	0.825
35	Severe symptoms of cholestasis	Proton-pump inhibitors	No	Heart failure, hepatic steatosis and failure, hypertension	Yes	0.63	0.71	1.05	2950	0.83667
36	Liver transplantation	No data	No data	No data	Yes	0.64	0.88	1.1	2600	0.9
37	Liver transplantation	Prograph, Augmentin MFF, prednisolone	No	T2 diabetes mellitus, thrombosis of the portal system	Yes	0.79	0.84	1.03	2600	0.928
38	Left lung nodule biopsy	-	No	Hypertension, diverticular disease, nephrolithiasis, atherosclerosis,	Yes	1.03	0.98	1.09	2350	0.908
39	Liver transplantation	Vancomycin, analgesics, anti-encephalopathic drugs, anti-coagulants	No	Renal failure, ascites, hemorrhagic diathesis	Yes	0.88	0.91	0.99	2600	0.772
40	Progressive liver damage	Propofol, norepinephrine, fluconazole	Yes	No	No	0.97	0.91	1.04	2950	0.9075
41	Pulmonary embolism	-	No	Extensive ulceration of the duodenal bulb, gastric hernia, liver cyst, diverticular disease	No	0.98	1.07	1.11	2350	0.744
42	Liver transplantation due to toxic damage	Sufentanil, midazolam, norepinephrine, colistin,	Yes	No data	No	1.0	1.2	1.15	2600	0.76
43	Primary sclerosing cholangitis and chronic liver failure	Analgesics, norepinephrine	No	Kidney failure	No	1.09	1.16	1.24	2950	0.893
44	Liver transplant	Analgesics	No	Esophageal varices, anemia, chronic pancreatitis, hypertension, diabetes mellitus	Yes	0.82	1.07	1.14	2950	1.07
45	Rectal perforation	Analgesics	No	Cirrhosis	Yes	0.83	1.07	1.09	2950	0.793
46	Acute kidney injury and tacrolimus poisoning	Diuretics, meropenem, diflucan	Yes	Depression, acute renal failure, hypertension, alcohol dependence syndrome	Yes	0.8	0.98	1.15	2950	0.75
47	Cirrhosis and primary sclerosing cholangitis	Pressor amines	Yes	Esophageal varices, Graves’ disease	Yes	0.64	0.78	0.82	2950	0.723
48	Inflammation and deterioration of kidney function	Tacrolimus, pressor amines, propofol	Yes	No	No	0.88	1.28	1.31	2600	0.873
49	Hepatic encephalopathy	-	No	Portal hypertension, splenomegaly, thrombocytopenia, chronic pancreatitis, diabetes mellitus	Yes	0.67	0.89	0.68	2350	0.572

**Table 2:** The association between the presence of factors potentially affecting magnesium homeostasis and the serum magnesium concentration preoperatively, 24 h postoperatively, and 48 h postoperatively.

Factors Potentially Affecting Magnesium Homeostasis	Preoperatively	24 h Postoperatively	48 h Postoperatively
Surgery	*p* = 0.29	*p* = 0.88	*p* = 0.09
Comorbidities	*p* = 0.11	*p* = 0.99	*p* = 0.88
Intravenous administration of sedatives	*p* = 0.55	*p* < 0.05	*p* < 0.05
Intravenous administration of diuretics	*p* = 0.11	*p* < 0.05	*p* < 0.05
Intravenous administration of antimicrobials	*p* = 0.57	*p* = 0.6	*p* = 0.43
Dialysis	*p* = 0.13	*p* = 0.65	*p* = 0.78

## Data Availability

The data presented in this study are available on request from the corresponding author. The data are not publicly available due to ethical reasons.
